# The Role of Artificial Intelligence in Nutrition Research: A Scoping Review

**DOI:** 10.3390/nu16132066

**Published:** 2024-06-28

**Authors:** Andrea Sosa-Holwerda, Oak-Hee Park, Kembra Albracht-Schulte, Surya Niraula, Leslie Thompson, Wilna Oldewage-Theron

**Affiliations:** 1 Department of Nutritional Sciences, Texas Tech University, Lubbock, TX 79409, USA; andrea.sosa@ttu.edu (A.S.-H.); surya.niraula@ttu.edu (S.N.); 2 College of Health & Human Sciences, Texas Tech University, Lubbock, TX 79409, USA; oak-hee.park@ttu.edu; 3 Department of Kinesiology & Sport Management, Texas Tech University, Lubbock, TX 79409, USA; kembra.albracht@ttu.edu; 4 Department of Animal and Food Sciences, Texas Tech University, Lubbock, TX 79409, USA; leslie.thompson@ttu.edu

**Keywords:** artificial intelligence, AI in nutrition, AI-nutritionist, chatbot, artificial intelligence and dietary assessment

## Abstract

Artificial intelligence (AI) refers to computer systems doing tasks that usually need human intelligence. AI is constantly changing and is revolutionizing the healthcare field, including nutrition. This review’s purpose is four-fold: (i) to investigate AI’s role in nutrition research; (ii) to identify areas in nutrition using AI; (iii) to understand AI’s future potential impact; (iv) to investigate possible concerns about AI’s use in nutrition research. Eight databases were searched: PubMed, Web of Science, EBSCO, Agricola, Scopus, IEEE Explore, Google Scholar and Cochrane. A total of 1737 articles were retrieved, of which 22 were included in the review. Article screening phases included duplicates elimination, title-abstract selection, full-text review, and quality assessment. The key findings indicated AI’s role in nutrition is at a developmental stage, focusing mainly on dietary assessment and less on malnutrition prediction, lifestyle interventions, and diet-related diseases comprehension. Clinical research is needed to determine AI’s intervention efficacy. The ethics of AI use, a main concern, remains unresolved and needs to be considered for collateral damage prevention to certain populations. The studies’ heterogeneity in this review limited the focus on specific nutritional areas. Future research should prioritize specialized reviews in nutrition and dieting for a deeper understanding of AI’s potential in human nutrition.

## 1. Introduction

Nutrition research seeks to investigate the relationship between health and diet in communities and at the individual level [1]. Nutrition practice (providing what is essential to enhance growth, development and prevent chronic diseases) and research are increasingly dependent on AI in terms of diagnoses, predictions and data explanation [2]. Diet and physical activity levels and the prevention of diet-related diseases are some of the areas that are involved in nutrition research [3]. AI has the potential to address most nutritional concerns, such as the identification of the causes and the potential treatments that are associated with cardiovascular diseases, diabetes, cancer and obesity [4]. AI can help us better understand more complex connections between food and health [5], including the effects of the lack of a healthy diet [6].

Due to its impact on human health, nutrition research is vital [1]. Nutritionists may provide nutrition education and guidance, and meal planning advice [7], whereas a dietitian may provide management of medical conditions, such as allergies, eating disorders, diabetes and kidney disease (though not limited to these), to ensure an individual’s nutritional requirements are met [8]. Nonetheless, these services are drastically shifting because of the transformative technologies that encompass patients’ records, chatbots and artificial intelligence (AI) to meet health issues [9].

A great number of AI applications are currently in use in high-income countries supporting healthcare. Is estimated that by 2026 about USD 150 billion will be saved in healthcare in the United States due to the implementation of AI applications [10]. Similarly, AI can help to enhance efficiency in community healthcare in disadvantaged communities [11], where patient care frequently faces challenges [12].

### 1.1. Defining Artificial Intelligence

*Computing Machinery and Intelligence* was an article published by Alan Turing in the 1950s. He discussed the process of building intelligent machines and how to test their intelligence. Until now, this test has been known as the “Turing test”, and it is a standard to determine the level of intelligence of artificial systems [13]. Nonetheless, it was not until 1956 that the term “artificial intelligence” was adopted at the AI conference in Dartmouth College, where topics such as the use of machines to mimic human intelligence were thoroughly examined initiating AI [13].

Over the past decades, different definitions of AI have emerged. Defining AI is challenging given the complexity of the subject. Several attempts to define AI have been made, and the definitions have been criticized, failing to achieve agreement [14]. In his paper, *What is Artificial Intelligence?* (1955), McCarthy [15] defines AI as “the science and engineering of making intelligent machines, especially computer programs. It is related to the similar task of using computers to understand human intelligence” (p2). The definition of AI requires understanding of its multidisciplinary character given that its conceptualization differs among fields [14] and it contributes to different scientific and engineering areas [16]. The term AI is adapted to individual interests, regardless of the area in which it has been used [17]. Thus, considering all of its uses and applications, defining AI becomes unfeasible [18].

Human intelligence and AI are concepts that cannot be used interchangeably [19]. However, the distinctions and similarities between humans and AI are being widely debated [20]. The term intelligence is applicable to both, AI and humans, because it refers to problem-solving and environmental adaptability [21]. Nevertheless, digital machines possess a distinct operating system that displays different cognitive skills from human biological skills. Human cognitive functions are limited (e.g., working memory capacity, speed in reading and calculation, and memory loss over time), as well as the information that can be processed, whereas AI operates with minimal limitations [20]. A summary of the types and branches of AI is provided in Table 1.

Not having a clear definition brings confusion and misunderstanding. Currently, with regard to research, it is not possible to anticipate a broadly recognized definition of AI [22].

During its evolution, AI has gone through three different development stages: an early stage, from 1956 to 1980, followed by an industrialization stage from 1980 to 2000, and, lastly, an explosion stage, starting in the 2000’s until now [23], and increasing markedly in 2022 with the launch of ChatGPT 3.5 [24].

**Table 1:** Types and branches of artificial intelligence.

Artificial Narrow Intelligence (ANI) [21]		Artificial General Intelligence (AGI) [21]		Artificial Super Intelligence (ASI) [21]	
Referred to as “weak AI”.		Referred to as “strong AI”.		Focused on AI building other AI machines.	
Simplest type of AI.		A machine score ≥70 is classified as intelligent based on the Turing test.		Meant to outsmart humans by exceeding human cognitive competencies.	
Can carry out specific tasks and cannot execute any other job that was not given to them.		Branch of AI that is currently being used by scientists.		A theoretical model because ASI has not surpassed humans.	
The most logical form of AI that people can use.		Can perform several tasks.			
**Branches of artificial intelligence**					
Natural Language Processing (NLP) [25,26,27,28,29]	Machine Learning (ML) [30,31,32,33]	Deep Learning (DL) [34,35,36,37,38]	Generic Algorithm (GA) [39]	Generative Pre-trained Transformer (GPT) [24,40,41,42]	Latent Dirichlet Allocation (LDA) [43]
Understands verbal and non-verbal communication like humans.	Mimics human learning process.	Automates a significant portion of the process and removes human intervention.	A problem-solver, used to find solutions with no need of additional information.	Responds like a human from the information received.	Discovers and identifies topics withing a large set of documents.

### 1.2. Applications of Artificial Intelligence

The rapid evolution of AI and its algorithms capable of understanding complex interactions has given AI the potential to offer different applications in healthcare domains [5]. For example, applications in medicine, such as AI digital technologies, are being integrated, especially in radiology, cardiology, oncology, pathology and dermatology [44] to detect anomalies based on image recognition [45]. Portion size and calorie estimation can also be performed using image recognition by imitating human thinking. Furthermore, AI can analyze information that does not rely on humans or human self-reported data [46] like 24 h dietary intake [35]. AI is needed increasingly to obtain meaningful outcomes because it has the capacity to analyze extensive data sets, especially in nutrition where a considerable amount of data are produced [47]. Nevertheless, AI applications are not well known in the nutrition field [6]

AI applications may be adapted and applied in nutrition, but they need to be investigated [44], since AI is bringing important changes shifting the way nutrition is currently delivered, from the use of conventional methods to the use of more sophisticated software to assess body weight, food intake, diet-related diseases, and of cutting-edge data storage systems to meet current demands using mobile applications, chatbots [9], and image recognition for dietary assessment [46]. However, concerns about privacy, integrity and accuracy when using AI still arise [30].

To the best of our knowledge, there is no research that specifically considers the areas where AI in nutrition research is being applied, its benefits and drawbacks. Thus, the purpose of this article is four-fold: (i) to investigate the role of AI in nutrition research, (ii) to identify the areas of nutrition in which AI is being used; (iii) to understand the potential impact it may have in the future, (iv) to investigate possible concerns about the use of AI in nutrition research.

## 2. Materials and Methods

A summary of the studies that were selected is described in Table 2. The studies are organized according to the type of AI that was used.

This review considered articles from 2000 to 2023 considering the current explosion stage of AI. Articles were searched across eight databases: PubMed, Web of Science, EBSCO, Agricola, Scopus, IEEE Explore, Google Scholar and Cochrane. Permutation was performed using terms such as *Nutr, *bot, *chatbot in combination with Boolean operators (AND and OR) and search strings. Studies where AI was directly applied in nutrition, studies where AI was used for nutritional assessment and to enhance lifestyle (nutrition-related), and where AI was used for human nutrition were considered. The articles selection was not limited to the United States. Animal studies and studies in any other language that was not English were excluded. A description of the inclusion and exclusion criteria for the studies’ characteristics is provided in Table 2.

**Figure 1:**
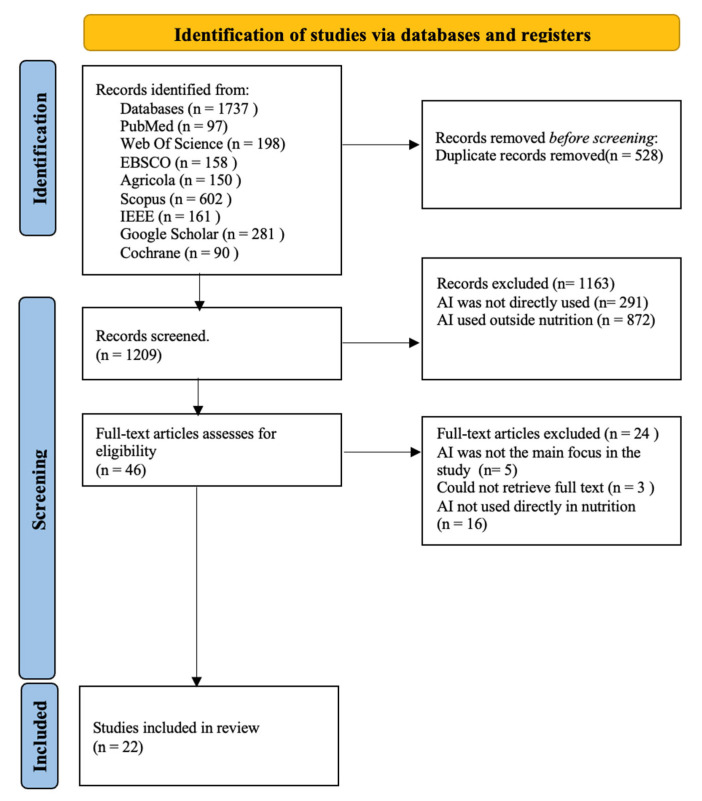
PRISMA flow diagram.

All the collected references were imported to Excel for duplicate removal. For the initial screening phase, titles and abstracts were selected based on the eligibility criteria in Table 2. If they met the criteria, they proceeded to the full-text review phase and if the inclusion criteria were met, eligible studies were assessed by their quality and included in the review, as shown in Figure 1. The information that was extracted from each study included author, year of publication, aim and topic, study characteristics, findings, limitations, type of AI used, and the nutrition area to which AI was applied. Additionally, observations were made for each study.

### Quality Assessment

The quality of the articles included in this review was assessed by two authors based on the quality assessment framework provided by Kitchenham et al. [48]. The quality assessment consisted of 12 questions (e.g., Is the paper based on research? What research method was used? Is there a clear statement of the aims of the studies?); if the question was fully, partially or not answered, a score of 1, 0.5 or 0 was assigned, respectively. A maximum score of 11 was attainable, considering that one of the questions asked about the research method only, and did not require a score. Interrater reliability over 97% was achieved.

## 3. Results

Our findings indicate that the primary role of AI in nutrition is mainly focused on providing dietary assessment, and to a lesser extent malnutrition prediction, lifestyle interventions and diet-related diseases.

A total of 1737 articles were retrieved, of which 22 met the inclusion criteria. In terms of quality, the maximum score (greatest quality) was 11 and the lowest score was 6.5 (still above the average score of 5.5). A total of 22 articles were considered eligible and included in the review article, with their characteristics shown in Table 3. A total of 22 studies published between 2019 and 2023 were included in the review article.

### 3.1. Description of the Included Studies

**Figure 2:**
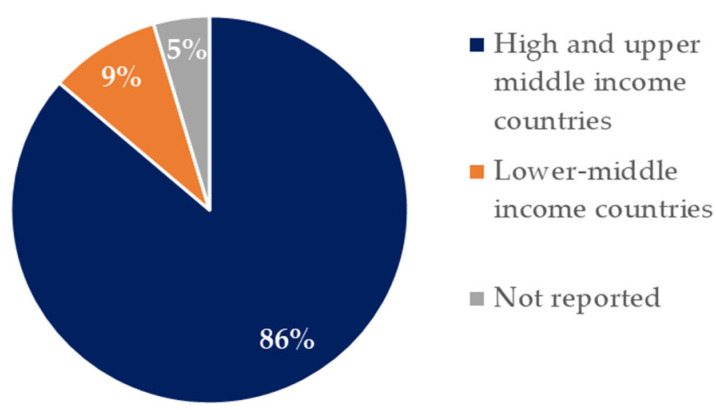
Studies distribution included in the review by country income classification.

Figure 2 shows the distribution of the studies that were included in this review by country classification according to the World Bank [49]. Eighty six percent of the studies are derived from high- and upper-middle-income countries, 9%, are from lower-middle-income countries. Only one study (5%) was not reported. Five were studies from the U.S. [25,28,30,50,51], two from Australia [26,27], three from China [34,35,42], two from Switzerland [29,36], one from Ghana [39], one from Canada [31], one from France [38], one from Brazil [43], one from India [32], one from Poland [40], one from Turkey [41], one from Japan [33], one from the UK [44], and one not reported [24].

The studies that were included in the review were categorized according to the area of nutrition where AI was used. A summary of the studies’ characteristics, findings and limitations was also performed, as shown in Table 4.

### 3.2. Applications of AI in Nutrition

**Figure 3:**
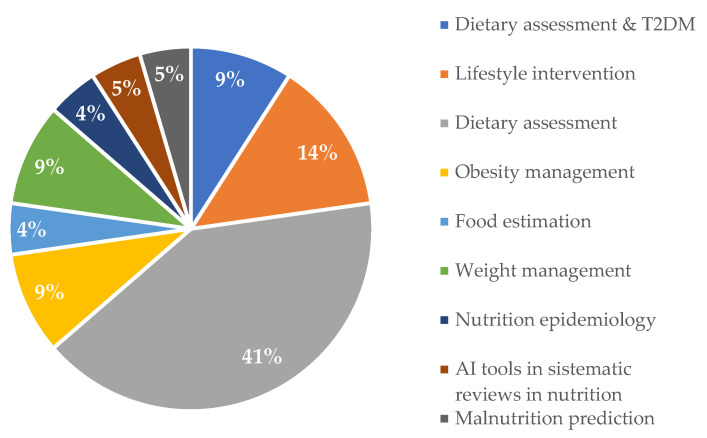
Nutrition areas in which the included articles used artificial intelligence.

Figure 3 shows the nutrition areas in which the included articles used AI. Most of the studies were focused on providing dietary assessment [24,25,29,30,32,36,37,38,40,44], followed by lifestyle interventions [26,27,28], and dietary assessment specifically for people with T2DM [25,42]. AI application to weight management [33,35] and obesity management [39,41] were represented by the same percentage.

### 3.3. Overview of the Studies

The number of the studies according to their focus and the area of nutrition to which AI was applied are presented in Table 3.

**Table 3:** Overview of the studies’ characteristics.

Five Descriptive Studies (22.73%) [24,30,32,40,44]	Nine ObservationalStudies (40.9%) [31,33,34,35,36,38,39,42,51]	Two Pilot Studies (9.09%) [26,50]	Two SystematicReviews (9.09%) [28,43]	Four Mixed Studies (18.19%) [25,27,29,41]
The impact of AI on dietitians. The use of ChatGPT in dietary planning and the accuracy of the diets. The benefits of AI for those who do not have access to dietitians.	Bariatric care knowledge. Portion size estimation. Body weight prediction. Malnutrition prediction. Relationship between food and CVD.	The use of AI to change lifestyle. Evaluation of food quality through crowdsourcing images.	The use of chatbots and their impact on physical activity, weight management and change in eating habits.	AI and its capacity for being culturally adapted. Obesity management. Weight loss and lifestyle intervention. T2MD management.

### 3.4. Testing Stage and Type of AI Used

The studies that used natural language processing (NLP) have the potential to improve nutrition through diet recommendations tailored to the user. One study using NLP relied on voice instead of written interaction to culturally adapt it and to make it user-friendly for those with low literacy and low technological skills [25]. The success of the voice-based AI ranged from 76% to 87% based on 150 conversations. The accuracy of AI’s recommendations when considering physical and socioeconomic status reached 100% [25] The remaining studies used text interactions [26,27,28,29]. A weekly analysis carried out for 12 weeks showed that the virtual-health assistant asked the right questions with 97% accuracy. However, when answering questions that it was not previously programmed for, it provided correct answers only 20% of the time [26]. A follow-up study was conducted, and the virtual-health assistant provided portion size recommendations based on the Mediterranean diet. It also helped to set up goals for physical activity, seeking to successfully engage participants in a program that was intended to increase physical activity and to make dietary changes. Participants lost 1.3 kg and their waist circumference was reduced by 2.1 cm in a 12-week period [27]. Most of the chatbots use persuasion and relational strategies to interact with users [28]. AI was also evaluated in terms of usability and the possibility to provide nutritional knowledge. It obtained a score of 87/100 in ease of use, 5.28/7 for satisfaction and was also perceived as reliable (5.5/7) to provide nutrition information [24].

The studies that used machine learning (ML) [29,30,31,32] were mainly focused on prediction in different areas of nutrition, and body weight was one of them. A model was developed using 3-year health data from 55,000 Japanese. Once the model learned the data tendency of individuals according to their records, it predicted body weight changes for the subsequent three years, by also identifying lifestyle behaviors; making it suitable in clinical practice. The accuracy of the model was evaluated with the root mean square error = 1.914, showing a similar performance to that of the multiple regression model which was 1.890 [33]. Similarly, ML was used to develop a prediction model in nutrition epidemiology to understand the relationship between nutrients and cardiovascular disease (CVD). With a data set of 12,130 people, dietary information and some other components, such as demographic and socioeconomic status, the model was able to predict nutritional variables linked to CVD. This CVD risk prediction model is equal or superior to other current tools used for the same purpose, with an area under the receiver-operating characteristic curve (AUROC) of 0.821 [31]. This indicates that the model has the capacity to differentiate between categories. A higher AUROC denotes a better prediction [52]. Another study involved creating an AI-based dietitian specifically for diet prediction and recommendation based on anthropometric measurements and demographic information provided by the user, which is the same information a patient would provide to a nutritionist [32]. ML along with technologies, has facilitated more complicated analyses.

Deep learning (DL) can recognize speech and images. Six studies that used DL focused on food estimation, dietary assessment, weight management and malnutrition prediction [34,35,36,38,50,51]. Four of the six studies using DL utilized it for dietary assessment. The 24 h recall has been acknowledged as the gold standard to report dietary intake; to lessen the burden an app was developed to facilitate self-reported dietary intake by either texting or speaking. When this app was compared (after 5 days of use) to the 24 h recall (two phone calls on different days), no significant difference was found between the two tools. The results were as follows: protein% (mean 16 vs. 17), fat % (mean 35 vs. 36), carbohydrates % (mean 50 vs. 50) and energy intake (2092 kcal/d vs. 2030 kcal/day) [35].

A different way to assess dietary intake was proposed by utilizing pictures of meals before and after patients had eaten; with the aim to prevent malnutrition among older patients in a hospital. Macronutrient intake was estimated. The AI dietary system surpassed the macronutrients estimation when compared to that of the nurses in a hospital who followed the hospital’s procedure compared to the control (two dietitians and a medical student). The AI system’s error for macronutrient and energy intake was less than 15% and 11.64%, respectively, whereas the nurses’ error was over 30% for macronutrient intake and 31.45% for energy. Furthermore, the AI system can generate the output almost instantly [36].

DL has the potential to predict malnutrition [51], assessing dietary intake using a smartphone by measuring leftovers using before and after pictures, estimating the patient’s food intake with a higher accuracy when compared to trained personnel [38]. DL can also be employed on a bigger scale to assess the nutritional quality of restaurant food using image recognition. Additionally, a nutrition index can be developed to evaluate the quality of food offered in areas where healthy food and food access are limited [50]. Similarly, the genetic algorithm (GA) has shown promise in food prediction to meet calorie intake of macro- and micronutrients, making it suitable as a future tool for obesity control [39].

ChatGPT was tested in terms of diet recommendations, accuracy and safety. When a fictitious woman asked for a diet telling ChatGPT about her food allergies, ChatGPT failed to recommend diets not having allergens in 4 of the 56 meals. A diet deficient in energy was selected on purpose to validate if the model would provide nutritional advice—no warning was generated, and energy was also miscalculated in some of the meals. For accuracy of the results the ChatGPT diets were assessed by a dietitian who had completed postgraduate studies in nutrition. It is worrisome that ChatGPT may provide misleading information to non-experts in nutrition. However, the AI diet recommended following dietary guidelines [40]. In contrast, another study asked ChatGPT to formulate a diet for a person with T2DM and the model was able to provide lunch recipes aligning with the recommendations of the American Diabetes Association (ADA). Similar results were given when the model was asked to provide a diet for a patient with hemodialysis; the recommended diet was aligned to the patient’s profile. However, when the questions were asked again, different answers were provided with incorrect dietary plans for the patients [24].

ChatGPT’s potential as an AI-based nutritionist was tested in different settings to evaluate its proficiency regarding T2DM. In terms of nutritional knowledge, ChatGPT (60.5%) and ChatGPT4 (74.5%) proved to be accurate. Additionally, its knowledge of a ketogenic diet was assessed with a set of 28 questions, showing an overlap score of 80.7 between ChatGPT and the experts. The evaluations were as follows: excellent (48.81%), acceptable (47.62%) and unacceptable (3.57%). Also, ChatGPT successfully passed the Chinese Registered Dietitian Exam and demonstrated that it can be compared to a registered dietitian in terms of examination and in clinical settings for predicting diseases such as CVD and obesity [42].

Latent Dirichlet Allocation (LDA) is a field of AI that is used to discover and to identify themes or topics within a large set of documents, enabling analysis and summarizing information that would be impossible for humans to analyze. Hence, it has been utilized to conduct systematic reviews in the field of nutrition. Among the topics detected by LDA, 75% were related to dietary assessments and diseases and the remaining study (25%) focused on sports nutrition. Analyses were limited, given the number of retracted articles [43]. It was found that AI can enhance healthcare by providing nutritional advice and that the rapidness of the results might benefit users. Hence, other uses in this area are expected to be developed. It is important to keep in mind that AI might introduce bias because it is trained by humans. Thus, the use of AI should be carried out with caution [44].

Table 4 provides a summary of the included articles in the review.

**Table 4:** Summary of studies.

Lead Author, Year & Country	Aim & Topic	Study Characteristics	Findings	Limitations	Type of AI	Area in Nutrition	Observations
Maharjan, B. (2019) USA [25]	The use and development of an AI tool that is culturally adapted for Native Americans with low computer/technological skills to support diabetes management using people’s voice.	Feasibility study. AI voice-based success range based on 150 conversations = 76% to 87% Accuracy regarding recommendations considering physical and socioeconomic status = 100%.	Preliminary results showed that Alexa was able to accurately count calories and provide nutrition education. Having the potential to improve health among the target population by recommending meals using ADA’s guidelines.	AI has to learn the needs of the specific target populations to culturally adapt it to other specific groups.	NLP	Dietary assessment for patients with T2DM.	Health improvements, validation and patient’s satisfaction using this technology is yet to be determined.
Davis, C.R. (2020) Australia [26]	To evaluate the performance of AI health assistance, and to verify participants’ adherence to physical activity, diet and their engagement	Pilot study Single-arm repeated measures for 12 weeks. *n* = 28 Adults 45–75 y/o Accuracy of answers provided to the users = 97% Accuracy of answers for which it was not trained = 20% Adherence (step and food serving goals) = 91%	Paola (the virtual health-assistant) was successful in behavior change. However, she could not answer questions beyond what she was trained for.	The sample size of the study was too small. Due to data loss, other questions (not related to what AI was trained in) could not be evaluated. Men are underrepresented; thus, the results cannot be generalized. The platform used to launch Paola had issues with 10 min time-outs	NLP	Lifestyle intervention	Paola also provided educational videos and different recipes. Users had weekly exchanges with her for data entry and to obtain her feedback based on their entry
Maher, C.A. (2020) Australia [27]	To test the recruitment and retention of a physical activity program that was also based on a Mediterranean diet. The program was delivered by an AI virtual health coach.	Interventional *n* = 31 Adults 45–75 y/o Participants lost 1.3 kg and waist circumference was reduced by 2.1 cm in 12 weeks	The virtual-health assistant (Paola) successfully delivered a lifestyle intervention program helping to lose weight and to increase participant’s physical activity. AI virtual coach has room for improvement regarding its connection to people in terms of emotions.	The study was not randomized and the follow up was limited	NLP	Lifestyle intervention	Participants showed enthusiasm using a virtual-health coach. This technology may be used in other nutrition areas, such as weight loss or diabetes management
Oh, Y. (2021) USA [28]	To assess the characteristics of chatbots in terms of conversation and function, and to investigate if chatbots interventions were successful in lifestyle changes (healthy eating, exercise, weight control) and health-related outcomes.	Systematic review. *n* = 9. 5 studies found chatbots had positive outcomes in physical activity. 1 study showed the intervention group reported the intention to eat less meat. Chatbots’ communication was text-based. Persuasion and relational strategies were used by chatbots. Interventions between 1 week–12 weeks with an age range = 15.2–56.2 y/o	Chatbots have the potential to change lifestyle and improve access and effectiveness to personalized nutrition.	Sample sizes used by the studies were too small; thus, it is difficult to draw conclusions on employment of chatbots to deliver lifestyle changing programs.	NLP	Lifestyle intervention	Studies did not evaluate side-effects nor the possible harm that users may encounter when using them. Chatbots should be used with caution, and conversations should be monitored to avoid harmful effects.
Beyeler, M. (2023) Switzerland [29]	To evaluate the usage of a health bot (HB) and how it is perceived by patients receiving bariatric treatment.	Mixed methods approach. Observational. AI usability = 87/100 Usefulness 5.28/7 Satisfaction 5.75/7 Learnability 6.26/7 Reliable nutrition information 5.5/7.	The health bot (HB) was assessed by its response to nutrition-related questions. HB was well accepted among patients, and they found it easy to use and understand. Participants had access to useful information through the HB. However, concerns about the replacements of dietitians, personal information and the privacy of the questions were brought up by the participants.	The sample size of the study was too small. AI used for dietary assessment should not be used without supervision of a healthcare professional due to the potential misinterpretation of the HB answers. HB may exclude people with no or limited access to digital resources, and limited literacy. Making an HB easy to use should be considered.	NLP	Dietary assessment	The HB was not meant to replace consultation; instead, it was meant to be used between consultations with a dietitian.
Limketkai, B.N. (2021) USA [30]	A review of new technologies (apps, wearable devices, and AI remote nutrition assessment) and their integration in clinical nutrition and patient care.	Descriptive study Wearable devices help users to engage. Currently there are ~165,000 apps related to health and wellness and 10,000 are for weight loss and dietary purposes. 58% of the population in the U.S have downloaded an app related to health. 83% of dietitians use mobile apps in clinic.	AI-based apps and wearables devices are used by clinicians since they can be used for diet optimization and to find eating patterns, given their real-time data collection. Smartwatches (e.g., Apple watch, Kardia band) have been approved by the FDA for some health uses, shifting from wellness devices to a more medical focus.	Wearable devices are still being developed, as algorithms cannot fully differentiate between different type of foods, portions, and backgrounds. Some technologies that measure body composition have not been tested in clinical trials; thus, the accuracy of the results needs to be assessed.	ML	Dietary assessment	Some apps that offer measurements such as sleeping patterns and heart rate require a monthly subscription and a smartphone. These emerging technologies in clinical nutrition are still in their infancy and need further investigation. There is concern about the information generated and its use in medical decision making.
Morgenstern, J.D. (2022) Canada [31]	To create a machine learning prediction model, and to evaluate its efficacy in examining the connection between food intake and CVD risk.	Retrospective cohort Observational *n* = 12,130 with a 14-year follow-up duration. The model’s accuracy has an AUROC = 0.821	The most significant nutritional variables linked to CVD were caffeine, alcohol, supplements and sodium. Without the need of lab tests and anthropometric measurements.	Nutritional variables were used, employing one-time 24 h recall. A larger data set with more frequent dietary assessment is needed. A separate model for dietary variables vs. non-dietary variables is needed to confirm dietary information for CVD prediction.	ML	Nutrition epidemiology	No lab tests and anthropometric variables were used in ML models.
Murumkar, A. (2023) India [32]	To develop an AI-based dietician that acts like a real dietitian. It offers diets and diet plans focused on the individual.	Prospective descriptive study The expected outcomes include BMI calculation and diet recommendations based on anthropometric and demographic data. Alternative diets will be displayed if the user rejects the original diet.	Feeding AI with appropriate information, such as BMI, allergies, food preferences, physical activity, and type of job; AI has the potential to suggest eating plans according to the user’s need without having to pay for it.	The user will be uploading information (height, weight, allergies, etc.) which is self-reported.	ML	Dietary assessment	No dietitian intervention is encouraged.
Fujihara, K. (2023) Japan [33]	To build, develop and evaluate the ability of a ML model to predict variations in body weight over a 3-year period from medical examinations.	Observational. *n* = 55,000, with a mean age = 48 y/o and 67% males The accuracy of the model was evaluated with the root mean square error = 1.914, similar to the 1.890 of the multiple regression The model successfully predicted body weight change among adults over 3 years using existing data.	The system was able to develop 5 different formulas for body weight change prediction over a 3-year span. It successfully identified lifestyle factors that modified body weight. It has the potential to be used in weight management.	The model may not be generalizable because it was developed using a particular ethnic group. Diet and physical activity information used to build the system was self-reported. Environmental and socioeconomic factors were not considered.	ML	Weight management	5-year data were used to develop the model. Data for 50,000 individuals were used to train the model and 5000 to test it.
Yang, Z. (2021) China [34]	To mimic a dietician’s mental process using AI for food size estimation.	Observational *n* = 15,000 pictures (for training) The accuracy of the model for volume estimation = 86.7%	This technology can be applied to wearable devices for real food volume estimation.	It was assumed that the food on a plate can easily be detected from a real-world image that also contains other things (e.g., table, background of the picture). It relies on high-quality object detection to crop the food plate from image. The volume estimation was limited with the plate having only one type of food; whereas in real life, a plate of food has more than one item.	DL	Food estimation.	Current data sets are designed for food recognition, but not for food volume (portion size) estimation.
Taylor, S. (2021) China [35]	To develop an AI-based app to map foods on national (U.S) databases, for calories counting vs. a recommended method.	Method comparison study Observational Males and females ≥ 18 y/o *n* = 35 with 5 days of food intake record. COCO nutritionist was compared to the 24HR with no significant difference, showing similar results. Protein% = 16 vs. 17 Fat% = 36 vs. 36 Carbs% = 50 vs. 50 Energy (kcal/d) = 2092 vs. 2030	National databases combined with an intelligent app using NLP; can estimate energy intake with no significant difference when compared to the 24HR, which is considered the gold standard for dietary intake. This may be used for weight management. Although participants had the option to speak to the COCO nutritionist to enter their dietary intake; they preferred to type their entries.	COCO nutritionist has limited features (it does not include food photography). The 24 h recall was used; food intake may not reflect complete dietary consumption because it is self-reported. The sample size for preliminary data is small.	DL	Weight management and dietary assessment.	MIT reviewed COCO nutritionist’s data without having access to the 24 h recall. 24 h recall was analyzed using a food processor.
Papathanail, I. (2021) Switzerland [36]	To develop and evaluate an AI system that uses input images for energy and macronutrient intake before and after patient’s consumption.	Observational, *n* = 28 332 pictures were captured over the course of 32 days. AI’s error was less for macronutrient (<15%) and energy intake (11.64%) vs. the control (>30% for macronutrients and 31.45% for energy intake)	The system’s estimation of macronutrients intake performed better than the control (nursing staff and a medical student) in the hospital. The system provides better estimation for individual meal components. AI provides results almost instantly	Meals were not weighted; dietitians and the medical student visually estimated food percentage.	DL	Dietary assessment	This system may be used to prevent malnutrition by monitoring diet among hospitalized older patients
Chen, X. (2021) USA [50]	To assess restaurant nutrition at a big scale by using crowdsourcing food images and to develop a restaurant nutrition index (quality of food offered by the restaurant based on calories).	Pilot study (Hartford area) *n* = 75 restaurant pictures AI accuracy for image recognition = 75.1% vs. 94.7% (trained raters).	DL used the restaurants’ pictures to determine the quality (calorie-based) of their food. Restaurants offering foods with higher calories were found in areas with limited food access and less healthy food retailers. These results may be used in food environment inequality assessment.	The model could not identify pictures with several food items on the same plate—it was able to estimate only one; it could not identify portion sizes derived from the images. Some foods were not accurately identified. Results cannot be generalized to other geographical areas because crowdsourcing images were from food review websites from a particular area. Restaurants with no online presence were excluded. Young adults were most of the raters, and this might have influenced the type of foods that were reviewed.	DL	Dietary assessment and food environment detection	This tool is not meant to replace current dietary assessment methods, it should be used as a complementary tool only.
Van Wymelbeke-Delannoy, V. (2022) France [38]	To assess food consumption using an AI system that does not need human interaction to determine food leftovers in a hospital setting.	*n* = 149 dishes, with 22,544 different scenarios (pictures with different amounts of food on the plates) Observational. Food intake estimation accuracy = 57.8%	The FoodIntech project was demonstrated to be useful in picture gathering and estimating patient’s food intake by analyzing food leftovers in a hospital setting, providing instant results. With enough pictures the system can learn to recognize new foods.	The camera vision is limited; thus a 100% performance will not be achieved. AI struggles with certain food containers. Thus, food segmentation is hard to achieve. Trained staff are needed to take pictures with good resolution, lighting and clarity, for adequate dietary assessment.	DL	Dietary assessment	Food Intech was evaluated in a hospital setting, but it was not tested with the hospital’s patients. This might allow to determine whether the patient’s food intake and other factors, such as age, gender, and weight, are related to food intake.
Jin, B.T. (2022) USA [51]	To evaluate the ability of a malnutrition prediction model using longitudinal patient records.	*n* = 5.9 million Observational. AUROC (model’s performance evaluation) = 0.854–0.869 The model successfully identified between malnourished and non-malnourished patients using their saved records.	DL is accurate in malnutrition prediction by using patients’ longitudinal data. AI used 3 visits instead of using only the patient’s last visit information for prediction. Neither lab tests nor anthropometric measures are used in this model; (less data collection) was needed, relying only on its capacity of predictive diagnosis.	Patients with minimal records or no records were excluded. This may represent bias towards populations at higher risk.	DL	Malnutrition prediction	This model may be incorporated into current healthcare using demographic and diagnostic data. However, this model still needs to undergo clinical validation.
Sefa-Yeboah, S.M. (2021) Ghana [39]	To develop a mobile app for obesity management working both on mobile and on the web; providing personalized meal plans to meet people’s macronutrients and calories needs.	Observational, *n* = 30 (potential solutions) with different population for 40 days. The simulations were tested using different values (kcal) 1000, 1600, 2000, 2400, 2800 and 3000. Number of generations (rounds) = 500.	AI engine can be used for meals recommendation, and prediction to meet calorie intake for obesity management. It estimates energy intake by selecting the foods from the food record. It also shows how many calories are left to meet the calorie goal.	The system’s overall effectiveness is impacted by the limited method used to assess physical activity, which does not allow to estimate energy expenditure. Additionally, the system is limited to food selection for dietary intake.	GA	Obesity management	This system can also be useful for training those who are in the dietetics field.
Niszczota, P. (2023) Poland [40]	To assess the performance of ChatGPT on diet generation by investigating the precision and safety of 56 diets generated by ChatGPT.	Validation study *n* = 56 generated diets designed for 1 fictitious allergic woman. 4 out of 56 diets provided diets with allergens. No supplementation was suggested with a low D-level diet. No warning was displayed with calorie restricted diet.	ChatGPT can generate menus, but it is not always safe; it included allergens for a fictitious allergic woman. It also provided wrong calculations for portion sizes, and it could not provide varied menus, repeating the same food items. This study was carried out in Europe and sometimes the measurements were provided in American units. ChatGPT may mislead people with its dietary suggestions. However, it followed recommendations from different dietary guidelines.	This study used only one prompt (one interaction) instead of a series of interactions. Big language models cannot identify when they are providing wrong information.	GPT	Dietary assessment	Results show that AI can also be misused and needs human interaction to verify that the information provided is correct. In some countries (e.g., Italy) ChatGPT had limited access.
Arslan, S. (2023) Turkey [41]	ChatGPT’s potential for treating and managing obesity. Based on the patient’s progress and records, ChatGPT may modify its recommendations.	Letter to the editor Description of potential uses of ChatGPT in obesity management: personalized diets, it can track the user’s progress, and diets can be adjusted from that progress.	ChatGPT can offer personalized advice such as weight control, physical activity and nutrition and meet individual’s needs. Based on the patient’s progress, recommendations for weight management can be adjusted.	AI’s information might be biased, depending on the type of data that were used to train it. AI-systems do not have emotional intelligence like a human and do not offer emotional support. When GPT provides harmful and inaccurate information, it is not clear who is to blame and who is responsible.	GPT	Obesity management	AI in healthcare must be used with caution and ethical issues must be addressed, since AI systems operate without ethical and professional standards.
Sun, H. (2023) China [42]	To develop and validate an AI-nutritionist focused on T2DM.	Validation study ChatGPT (60.5%) and ChatGPT4 (74.5%) proved to be accurate in nutritional knowledge. Ketogenic diet knowledge = 80.7 with 48.81%, classified as excellent, 47.62%, classified as acceptable and 3.57%, classified as unacceptable An overlap of 94.87% between GPT and experts in recommended foods Answers were validated by expert dietitians. 23% of endocrinologists categorized pork as high glycemic food.	ChatGPT and GPT4 are competent to answer the Chinese Register Dietitian Exam and medical nutrition-related questions. It also identified food using pictures. Endocrinologists’ knowledge regarding nutrition might not be reliable. AI has potential to provide dietary assessment and meet the lack of dietitians in China.	The model was presented only with a limited set of questions that a patient may ask. One of AI’s limitations in the training process is that it can provide several answers for the same question; hence, focusing on specific questions might help to obtain more trustworthy responses.	GPT	Dietary assessment for people with T2DM	The model was not tested, nor has a pilot study been conducted. When testing is performed, the authors recommend reviewing the AI-nutritionist’s answers by a human within 48 h span to ensure no harmful/wrong information is provided to the patient, allowing this to be fixed.
Chatelan, A. (2023)Not specified [24]	To provide a guide regarding the potential hazards and benefits of using ChatGPT in clinical, academic and public health contexts.	Descriptive study ChatGPT was able to provide diets according to users’ need (e.g., a patient with T2DM and a patient undergoing dialysis). For a patient with T2DM the diet provided ADA’s recommendations.	Using ChatGPT has both opportunities and risks. It might be beneficial for people to obtain educational material for free (healthy eating, nutrition). However, ChatGPT is not always accurate and might provide harmful responses. Therefore, it should be supervised. Chatbots do not have soft skills, making it harder to replace RDs.	ChatGPT might provide nutritional advice and diets, nonetheless. It cannot provide emotional and psychological support. ChatGPT does not cite the information sources it uses to provide answers; making it hard to determine whether the sources are factual or not.	GPT	Dietary assessment	ChatGPT trainings are limited. It is not aware of information that happens thereafter (it was last trained in January 2022). Given the quick evolution of chatbots, its potential uses are hard to define.
Nunes-Galbez, N. (2022) Brazil [43]	To evaluate AI tools for conducting systematic reviews in the nutrition field	Systematic review, *n* = 4 75% of the studies were related to dietary assessments and the cause of diseases. The remaining study (25%) was focused on sports nutrition.	The publication dates range from 2015 to 2021. All the retrieved publications are from developed countries. The small number of studies shows that AI is still novel in systematic reviews in nutrition.	The studies that did not address any challenges could be useful when considering the use of these technologies. The review was limited by the number of studies that were included.	LDA	Use of AI tools in systematic reviews in nutrition.	Big data has resulted in an exponential growth in scientific papers. It is hard for scientists to conduct a systematic review without losing data. In consequence, the use of AI has been proposed.
Bond, A. (2023) UK [44]	To identify areas and applications in nutrition where AI might play a role.	Descriptive study DL may deliver dietary assessment that is comparable to or exceed that of a certified dietitian.	AI can be used to enhance healthcare by interpreting images, making prescriptions, and to provide nutritional advice. In a hospital setting, patients can benefit from these technologies instead of waiting for the dietitian. Other uses in healthcare are expected to be developed.	AI might be biased since it is trained by humans. Thus, training AI should be performed with caution. If not trained properly, AI might face opposition within healthcare. Ethical concerns and how AI deals with personal information are still complex.	DL, ML, NLP	Dietary assessment	Not losing control of AI and how it is used in healthcare is of extreme importance. Healthcare personnel should understand how to use AI

Abbreviations: AI: artificial intelligence; cm: centimeters; T2DM: Type 2 Diabetes Mellitus; apps: applications; FDA: Food and Drug Administration; 24HR: 24 h recall; y/o: years old; kcal: kilocalories; MIT: Massachusetts Institute of Technology; CVD: cardiovascular disease; AUROC: area under the receiver-operating characteristic curve; BMI: body mass index; ADA: American Diabetes Association; DL: deep learning; ML: machine learning; NLP: natural language processing; GA: genetic algorithm; GPT: generative pre-trained transformer; LDA: latent Dirichlet allocation.

## 4. Discussion

AI has many different definitions, equal in number to the subfields within AI itself. This diversity continues to grow over time [53]. Confusion and misunderstanding result from a lack of a clear definition of AI [22]. In simple terms, AI refers to how computer systems can do tasks that usually need human intelligence or intervention. Image and speech recognition and even decision making are some examples of computers performing human tasks [53].

This review found that: (1) AI in nutrition is based on data collection and data analysis mostly focused on providing dietary assessment and to a lesser extent malnutrition prediction, lifestyle intervention, and to understand the relationship between nutrition and health; (2) The future impact of AI may transform the accuracy of results by eliminating biases from self-reported data and make nutrition information and recommendations available to a greater extent; (3) AI can increase dietary and physical activity adherence; and (4) the use of AI brings potential harms regarding people’s safety, integrity, and ethical issues that cannot be ruled out.

It is worth mentioning that most of the reviewed studies are still in a developmental stage of creating AI-based tools. These studies often involve extensive testing to assess their performance and the accuracy of results before they can be implemented [54]. Thomas, et al. (2022) [2] describe a series of steps to validate AI in nutrition research as follows: goal and data description, AI fitness, establishment of criteria assessment, pre-processing of data, development and evaluation of the algorithm. Only one study had an intervention using a virtual-health assistant. Therefore, the lack of interventions in the reviewed papers is attributed to the early stages of AI tools’ development that are focusing on testing and adjusting AI’s functionalities as needed, before their implementation and evaluation in real-life interventions.

Given the nature of AI and the numerous cases in which it can be implemented in human nutrition, this review covers a wide range of studies and topics in nutrition research. This heterogeneity among studies makes it challenging to focus on specific subfields of nutrition and diet.

### 4.1. Dietary Assessment

AI is evolving quickly and has the potential to revolutionize the nutrition field, especially clinical nutrition. In this review, it was notable that dietary assessment is one of the main areas where AI plays an important role in the field of nutrition. Studies included in the current review indicated that the use of AI in dietary intake can be very beneficial [24,25,29,30,36,38,40,42,43,44,50]. When it comes to data collection in dietary assessment, time and accuracy are some of the limitations. Clinicians rely on questionnaires to document patients’ health status and collecting participants data can be very expensive and requires a substantial investment of time [55].

AI has demonstrated the potential to provide accurate results on dietary assessments, while eliminating the burden and biases associated with self-reported data entry by participants [36,56,57]. Wearable devices can collect the user’s data, which can be utilized as baseline data to keep track of people’s improvement regarding diet and physical activity. These apps help to keep users engaged by displaying immediate information. In the U.S alone, 58% of individuals who use phones have downloaded apps related to health and 83% of dietitians use them [30].

The COCO nutritionist, which combines speech understanding and maps foods is another clear example of how an AI-based health assistant was successful in estimating dietary intake without the burden that 24 h recall places on participants [35]. The 24 h recall is considered to be the gold standard for food intake assessment; the COCO nutritionist was as accurate as the gold standard itself, showing no significant difference between the two tools [35]. AI was also used in an app for dietary assessment in Ghana and it was found that the AI results were equally accurate as those obtained using the 24 h recall, showing the promising features of AI in dietary intake estimation [58].

There is a significant emphasis on AI functioning as a dietitian, including its ability to calculate portion sizes. Another way to assess dietary intake is by image recognition without relying on humans [34]. To provide a complete dietary assessment through image recognition AI detects, recognizes and estimates volumes and analyzes food’s nutritional content, with the flexibility to adapt it to wearable devices [34]. This type of dietary assessment has become more common. Agreeing with this, the review conducted by Tahir, G.A. and C.K. Loo [59] showed almost 67% of the evaluated studies employing image recognition for the same purposes. In contrast, and despite the potential AI has in dietary assessment, in their study, Chen et al. [50] revealed that AI could not identify more than one food item on the plate, which does not represent a real-life scenario. There are other problems that are not limited to the number of food items served on the plate, including issues related to the quality of the pictures (light and shadow interference) [60], and certain food and food containers posing greater challenges in terms of food recognition and segmentation compared to others due to different shapes, colors and food ingredients [38]. This mixture on the plate adds to the complexity for food identification. The authors recommended trained staff should be used to obtain high-quality pictures. Therefore, the intended purpose of having AI performing dietary assessment without having to depend on humans would be defeated. Hence, the justification for use of AI in dietary assessment is not yet conclusive and needs further investigation.

### 4.2. Dietary Adherence

In dietary assessment, adherence is very important. A growing body of evidence shows that a high level of adherence to a diet is a key factor in achieving successful weight management [61]. When it comes to following dietary guidelines, the adherence rate can be even less than 50% [37]. AI has demonstrated encouraging outcomes in increasing adherence in patients who utilize technology [62]. Such was the case for the AI-based health assistant Paola, who achieved a 91% adherence to the recommended diet and step goals for physical activity, by providing recipes, educational videos and weekly interaction. Adherence involves understanding diet, self-efficacy and obtaining support [63]. The reason why Paola was successful might be attributed to her addressing these characteristics.

Data from Medicare revealed significant rates of non-adherence among patients; 76% of them were not taking their corresponding medicine, but when an AI-app was used providing daily reminders and instructions regarding the drug dosage to patients who had had a stroke, 100% adherence was achieved [62]. Like Paola, AI had a one-on-one interaction with the user; thus, we can infer that the interaction and specific instructions are contributing to achieving higher adherence. Although for some, AI is limited to only provide dietary assessment without being capable of providing emotional support [24], others say that through artificial emotional intelligence AI can respond to human emotions because humas can express their emotions through non-verbal communication. Computing systems can recognize and detect emotions from speech and by detection of facial expressions [64], opening new research opportunities in clinical nutrition.

Adjusting to the specific needs of the user, AI can enhance the experience and as previously mentioned, the user’s adherence as well to dietary intake. For example, using a voice-based virtual assistance, AI was successfully adapted to Native Americans, facilitating interaction between the user and AI [25]. Which is of importance given that there are only a few models that have been trained on indigenous population [65].

### 4.3. Ethical Issues

Despite the high importance of ethical issues and concerns that are associated with the use of artificial intelligence, these were barely mentioned—just in two articles [41,44] that were included in this review.

AI developers should apply ethical principles when developing artificial intelligence models [66]. As of now, there are no polices, regulations, standards and governance regarding the use of AI [67]. Not having a specific definition for AI could have significant implications when it comes to legal and ethical aspects [14].

One of the main concerns regarding AI and ethics is bias. If the information that is being used to train the model is biased or is not complete, it is most likely that this will be retained within the data. For instance, AI algorithms trained to schedule medical appointments based on patients’ attendance, will schedule African Americans into the least convenient appointments or even worse; AI might not even schedule an appointment, without considering that African Americans tend to be late or skip healthcare appointments due to their socioeconomic status (but not limited to this) [68]. Similarly, it may represent the population having health insurance, and not consider those without it [69]. In a study conducted by Makhortykh et al. [70], gender and bias were assessed and it was found that in six different search engines that AI favored white human representation in Western countries; gender was less skewed. Bias might be the result of social inequity, error in measurements and overfitted models [69], which is when the algorithm is very close to the training data, causing failure to carry out its prediction task on new data [71]. To avoid this, data cleaning is essential before training AI [44].

Patient’s privacy should be a priority, considering that AI is trained using real world data (data pertaining to the health status of a patient) [72]. For example, 1.6 million personal records were obtained by Google for AI development without having the consent of the patients [69]. This should call for a comprehensive review of ethics now that AI is in its early stages.

Liability is another big concern, especially because technology advances faster than laws [73]. If AI misdiagnoses or does not function properly, determining liability is not clear, highlighting that the need for regulations is of extreme importance [69]. Lack of transparency and not being able to understand how AI makes decisions (i.e., Blackbox) is also worrisome because DL can make biased decisions that might affect humans in a negative way [74].

### 4.4. Implications for Research

The integration of AI in the field of nutrition research can revolutionize the way we learn about and do things, bringing opportunities to advance human nutrition. That is the case of dietary intake, which can be easily performed without bias, providing more precise results. On a more complex level, AI has the potential to analyze genetics, culture, lifestyle, and health conditions at once, and enable precision nutrition. This is where the integration of medical nutrition and nutrition research converges, being crucial for prevention and management of chronic diseases due to the lack of nutrition education that exists within medical nutrition [74]. It can help to achieve results in nutrition research in a more efficient way in terms of economic and human resources.

For developed countries, AI can contribute in several ways (e.g., pre- and post-surgery recommendations to combat obesity [29], and improvements in healthcare [10]). Considering that developed countries have data and records, it can predict diseases in advance using the patient’s medical records. In addition, the general population has more access to AI-based devices that can help monitor their conditions in real-time. This information can be shared with nutritionists and dietitians to assess the patient accurately.

Given the ability to predict and its versatility in the field of nutrition, artificial intelligence can be both beneficial and harmful when applied in developing countries. On one side, having AI in communities where medical facilities, economic restrictions, health insurance and basic needs are lacking, may have a great impact to prevent malnutrition and diet-related diseases, achieving one of the main goals in community nutrition, which is prevention. It can also facilitate nutrition education, recognizing cultural food preferences and suggesting meals that are culturally adapted. It can help in diet-related diseases and weight management by providing clear instructions and one-on-one interaction with the user. However, it can also damage those countries. As discussed, AI can be biased in several ways (e.g., economic status and race). For example, in one of the studies that was included, the exclusion criteria included not having an iPhone. The fact that most of the countries in this review are high- and upper-middle income countries and with global market interest expected to grow to USD 45.2 billion by 2026 [72], AI might lead to greater investment in developed countries than in those who do not have the means to navigate and implement AI, limiting developing countries opportunities even more. This highlights the importance of having clear and well-established regulations that also protect underserved communities and populations.

### 4.5. Limitations

A limitation of this study is the limited number of interventional studies. Additionally, the studies are still at the development stage. Therefore, no solid conclusion can be drawn about the safety of participants using AI, nor about the efficacy and accuracy of the results in nutrition derived from AI; these aspects need further assessment. Another limitation is the language of studies—studies that were not written in English were excluded.

### 4.6. Recommnedations

It would be beneficial to also address dietary quality along with dietary assessment to achieve a broader understanding of people’s nutrition, in order to carry out potential nutrition interventions and enhance people’s quality of life. Dietary quality, emotional support, and populations with low literacy who are also technologically challenged are some of the emerging opportunities for broader future investigations in the nutrition field using AI.

One of the biggest gaps in nutrition research using AI is properly addressing ethical issues, especially where AI is being looked at with the potential to function as a dietician. People should be confident of how their data will be stored, analyzed and used. Thus, we recommend transparency when working with AI in nutrition.

Moreover, this thorough review showed the need for more research, specifically on undernutrition, particularly in developing and low-income countries; this would allow to assess more directly AI’s potential impact and to obtain more generalizable outcomes on the use of AI in nutrition. However, caution should be exercised with the aim of protecting underserved populations from the aforementioned risks.

Due to the encountered limitations and the rapid pace of AI advances, we recommend focusing on future interventional studies for broader investigations that could provide more definitive evidence, to ensure that AI is not reinforcing inequalities and that AI’s recommendations in human nutrition are clinically valid.

## 5. Conclusions

We conclude that (1) AI in the nutrition field still lags and needs far more research compared to other fields, such as medicine; (2) health improvement, artificial intelligence validation, artificial intelligence accuracy and patients’ satisfaction in nutrition are yet to be determined; (3) clinical research is needed to determine the efficacy of interventions using artificial intelligence; (4) ethics are one of the main concerns about AI’s use; thus, they need to be considered to avoid collateral damage to certain populations. These issues remain unresolved; (5) the heterogeneity of the studies included in this review limited the focus on specific nutritional areas; thus, future research should prioritize specialized reviews in nutrition and dieting to provide a deeper focus and understanding on the promising potential of AI in human nutrition.

## Figures and Tables

**Table 2:** PICOS (population, intervention, comparison, outcome, study design).

Research Question	What Is the Role of AI in Nutrition Research?	
	Inclusion Criteria	Exclusion Criteria
Population	Studies involved in the human nutrition field, areas within the nutrition field.	Studies involved in any other areas that were not related to human nutrition.
Intervention	Studies where AI was used, applied, or implemented in nutrition, or where AI played a main role or was a key part of the research with impact in human nutrition and/or nutrition research.	Studies that did not use AI in their research, studies where AI did not play a main role nor was a key part of the research. Studies that did not focus on human nutrition.
Comparison	Not set	None
Outcome	AI’s impact in human nutrition, contribution in nutrition research, influence on decision making in nutrition, recommendation of AI’s implementation in nutrition research, prediction of nutritional status, dietary intake, and diet recommendations, how AI is being used in nutrition research.	Studies that did not use AI for human nutrition, studies reporting only AI’s algorithm without emphasizing the use of AI in nutrition.
Study Design	Not set	None
